# Using the LSTM Neural Network and the UWB Positioning System to Predict the Position of Low and High Speed Moving Objects

**DOI:** 10.3390/s23198270

**Published:** 2023-10-06

**Authors:** Krzysztof Paszek, Damian Grzechca

**Affiliations:** 1 Department of Telecommunications and Teleinformatics, Faculty of Automatic Control, Electronics and Computer Science, Silesian University of Technology, Akademicka 16, 44-100 Gliwice, Poland; 2 Department of Electronics, Electrical Engineering and Microelectronics, Faculty of Automatic Control, Electronics and Computer Science, Silesian University of Technology, Akademicka 16, 44-100 Gliwice, Poland

**Keywords:** UWB, IMU, positioning, position prediction, neural network, LSTM

## Abstract

Automation of transportation will play a crucial role in the future when people driving vehicles will be replaced by autonomous systems. Currently, the positioning systems are not used alone but are combined in order to create cooperative positioning systems. The ultra-wideband (UWB) system is an excellent alternative to the global positioning system (GPS) in a limited area but has some drawbacks. Despite many advantages of various object positioning systems, none is free from the problem of object displacement during measurement (data acquisition), which affects positioning accuracy. In addition, temporarily missing data from the absolute positioning system can lead to dangerous situations. Moreover, data pre-processing is unavoidable and takes some time, affecting additionally the object’s displacement in relation to its previous position and its starting point of the new positioning process. So, the prediction of the position of an object is necessary to minimize the time when the position is unknown or out of date, especially when the object is moving at high speed and the position update rate is low. This article proposes using the long short-term memory (LSTM) artificial neural network to predict objects’ positions based on historical data from the UWB system and inertial navigation. The proposed solution creates a reliable positioning system that predicts 10 positions of low and high-speed moving objects with an error below 10 cm. Position prediction allows detection of possible collisions—the intersection of the trajectories of moving objects.

## 1. Introduction

Developing economy, and thus industry, is primarily based on the automation of processes. A good example of this is the automation of the movement of objects (driving a vehicle), which is noticeable not only in industrial halls but also in road traffic. Both scientists and companies are genuinely interested in automated warehouses [1,2]. It can already be seen in such places where goods are transported by autonomous robots—automated guided vehicles (AGVs) [3,4]. This usually happens along strictly defined routes, but humans are not involved in the transport process.

Automation of transport is a crucial element of the future, where it will support (it has already been partially achieved) or replace people in the process of driving a vehicle. Companies already offer public transportation using autonomous vehicles [5]. The future of autonomous vehicles is also confirmed by works carried out not only in the field related to systems and positioning technologies but also to aspects such as timetable planning [6], ethical and financial considerations [7], and accessibility [8].

Many technologies provide additional information to drivers, allowing them to see things normally invisible to the driver, such as a vehicle in the blind spot. Radars, lidars [9,10,11,12,13,14], video analysis [15,16,17,18,19,20], ultrasonic and hall systems [21,22] observe and scan the vehicle’s surroundings and can provide information about the impending danger (i.e., the position of the object relative to an obstacle or another traffic participant is determined). They can also decide and act without the driver’s intervention, e.g., brake in an emergency or park the vehicle in the indicated place (active parking assist) [23]. These systems also allow the monitoring of the distance to the car in front, warning the driver about not keeping sufficient distance or automatically adjusting the speed to the current traffic on the road [24]. Nevertheless, these systems must “see” other traffic participants or an obstacle so that the superior system can react—thus, the object must be within the device’s field of view (FOV), which is usually limited vertically and horizontally. The information about the obstacle’s or moving object’s position must be known in advance so the system has time to make a decision (e.g., warn the driver or stop the vehicle). This is particularly important in highly urbanized areas or warehouse halls, i.e., in places where visibility for both drivers/operators and the systems scanning the environment (radars, lidars and vision systems) is limited and does not allow to protect moving vehicles against possible dangerous situations. Additionally, information about dangers has to be propagate and therefore vehicles must communicate with the environment and each other [25,26]. To ensure the most recent data, the system should be a real-time system in order to perform actions and respond to events within predictable and specific time constraints [27]. In positioning systems, defined time constraints allow to determine the possible displacement of the object from the position in which the sensors’ data were obtained to the place where the information about the position is available. Time constraints and the possible displacement associated with them are used as one of the parameters determining the minimum distance from objects to other objects or obstacles—the position uncertainty. Various rules are used to ensure a safe distance between vehicles by a driver. One of them—the three-second rule, says that the driver should maintain a minimum distance from the vehicle ahead, which is equivalent to 3 s of reaching this object at a given speed. This time may seem long, but it should be considered that the driver (human) needs time to react—the time (and therefore the distance) can be shortened for computer systems where the information processing time is shorter. However, the approaching object must still be visible.

As can be seen, systems capable of detecting a possibly dangerous situation will not always be able to detect and predict collision, and their most significant limitation is the requirement for visibility. Thus, for this task, another technology is required to determine the object’s absolute position in the local or global frame of reference so that it is possible to determine the trajectories of moving objects and their potential collisions. In cooperation with inertial navigation, the GPS is the most commonly used system to determine the absolute position [28,29,30]. However, the GPS signal is often distorted, reflected, attenuated, or utterly unavailable in urbanized areas, and inertial navigation system errors accumulate over time [31].

Ultra-wideband (UWB) is a rapidly developing technology increasingly used to position objects indoors. The UWB positioning system provides information about distances between network nodes—tags associated with the positioned object and the anchors (reference points). Then, the obtained distances are used to calculate the position in the trilateration process [32,33]. UWB technology has become a beneficial alternative to other systems (especially GPS) used in the automotive industry (in limited areas) due to its low energy demand, low costs, additional data transferability, and high communication security [34,35,36,37,38]. Due to many possible applications, the UWB system is not only an alternative but is also increasingly used with other systems that determine the position of an object (as a supplementary system), creating a cooperative positioning system [39]. The advantages of the UWB system, such as the accurate positioning or the possibility to communicate, make this system widely researched [40,41,42]. Still, one should always remember the system’s limitations, such as the frequency of acquiring positions (and connected with its displacement), as well as the possibility of communication errors or loss. Yet, this system is mainly studied for the positioning of slowly moving or static (at least at the moment of measurement) objects, so an indoor positioning system (IPS) is used. However, with the increase in the speed of the object and the decrease in the frequency of obtaining data from the system, the displacement becomes greater; therefore, position prediction becomes crucial and is even required within the context of real-time locating systems (RTLS). Additionally, the maximum distance between nodes is limited and depends on system configuration. For example, the range of DW1000 modules working in operational mode 2 is limited to 100 m; mode 5 is limited to 50 m (on the market, there are also modules with an amplifier that allow ranging over 150 m). In practice, the range is lower because of signal attenuation and disruption. For this reason, the usage of the UWB system in larger areas requires more UWB nodes. This, in turn, introduces new problems in scheduling communications to avoid collisions and to ensure the highest frequency of position acquisition. In the studies presented in this article, the movement of the object with an initial speed of up to 20 m/s is considered, which covers the use of the UWB not only as an IPS but also as an outdoor positioning system (OPS). The problem of displacement (resulting from delays in the positioning system mentioned above) can be solved by predicting the object’s position based on historical data from the positioning system or systems. In addition, position prediction allows continued positioning in situations where a temporary signal loss from the UWB system occurs (no data packet with position). Then, without prediction, it is impossible to determine the object’s absolute position in the local frame of reference, and thus, it is impossible to avoid dangerous situations. It may seem that at low speeds with which AGVs move, position prediction does not matter. However, the inertia of a vehicle carrying a heavy load (several tons), for which the braking distance will be long, should be taken into account.

Consider a situation where a vehicle moves along a narrow road surrounded by buildings (or a warehouse that is densely packed with racks). When approaching an intersection, the driver or autonomous system should ensure it is safe to cross the intersection. However, in highly limited visibility conditions, a driver or an assistance system may need a significant speed reduction or even stopping the vehicle (regardless of road markings) to ensure safety. The ability to predict objects’ positions and communication between vehicles is required to provide the biggest possible capacity of a road (or the fastest transportation of goods in warehouses) and to eliminate possible dangerous situations in this type of scenario. Knowing the future positions of the vehicles, it is possible to determine whether vehicles’ trajectories intersect and, if so, when and whether a dangerous situation may occur.

Prediction of the position of moving objects is, in other words, forecasting an event or feature value of a variable based on information from the past. Various methods are used to forecast the value of a variable, e.g., Kalman filter (KF), autoregressive integrated moving average (ARIMA) [43], or recurrent neural networks (e.g., LSTM) [44,45]. KF is widely used to estimate the position of an object. Its application involves the preparation of a mathematical model, which also allows for data fusion in multi-sensor systems. With the increase in data sources, the mathematical model becomes complex and often requires certain assumptions to simplify the model. Finding a direct relationship between the data is sometimes complicated or even impossible. The big challenge is to tune the noise covariance matrix, which tells how relevant the model is, i.e., what is the difference between the model and the actual motion of the object. ARIMA models are used for short-period prediction based on past data. This model is widely used for forecasting univariate problems—cannot capture dependencies between different data sources (variables). The vector autoregressive (VAR) is designed for multivariate problems—so it can consider multiple data sources and find the relation between them [46]. This model can be extended with exogenous variables (unmodeled inputs to the model) that are introduced to explain outcomes in the model (VARX—vector autoregression with exogenous series). The neural network-based approaches can find dependencies between a large number of variables without a complex mathematical model—so the way of data fusion is learned during training. The commonly used recurrent neural network for forecasting problems is LSTM. In the position prediction, the dynamic model can be unknown for LSTM in contrast to KF, where the model needs to be specified.

Undoubtedly, the use of the UWB system will make it possible to “see” other traffic participants, and the prediction of the object’s position using the LSTM network may lead to the determination of the trajectory of the objects and their possible intersection—a dangerous situation, solving the problem of limited frequency of acquiring positions and displacement during data acquisition and processing (which is especially important when the subject is moving in a dense environment and/or at high speed).

In positioning systems, the KF, which is often used to estimate (filter) positions based on various data from various technologies, comes in different versions. Data from inertial sensors and the UWB system are commonly fused in multiple ways using KF. In [47], the authors presented an interesting method that combines UWB and pedestrian dead reckoning (PDR) in different conditions of the density of UWB anchors and different visibility of positioning pedestrians. They prepared two Kalman filters for orientation and position, using dynamic and fixed variance estimation. The authors also emphasize that incorrect error quantification may affect the system’s filtering capacity (position estimation) for a longer time. The average position error of their method under line-of-sight (LOS) conditions with a high density of anchors is about 0.5 m. The authors of [48] presented the weaknesses of the Kalman filter and its various modifications (extended, unscented, and cubature Kalman filters) both in simulation and real tests. These include, among others, the lack of gross error resistance, dependency on the initial values, and problems with the linearization and stability of the algorithms. The RMSE of the position in simulated static measurements ranges from 42–191 cm, depending on the Kalman filter variant used. The authors proposed the use of a robust particle filter for which the RMSE is 40 cm in the same test scenarios, but according to the authors, the computation is complex and time-consuming.

Apart from traditional methods, machine learning methods are also used to increase the accuracy of determined positions. Their use is not always directly related to the object position, e.g., fingerprinting [49,50], but they have an impact on it, e.g., by detecting LOS/non-line-of-sight (NLOS) conditions [51,52,53] or even LOS/NLOS/multi-path (MP) conditions [54] or system error prediction [55,56]. In [57], the authors proposed the use of an LSTM network to determine the position of an object based on distances to 3 reference nodes. The distances used in the research were also obtained from the simulation environment of the UWB system, as well as the ground truth, which is difficult to obtain in other than simulation conditions. The accuracy of the proposed network was compared to other methods based on least square estimation, fingerprint estimation, maximum likelihood estimation and weighted centroid estimation algorithms, back propagation neural network, extreme learning machine, and recurrent neural networks. The authors indicated that their LSTM network approach allowed them to locate the object with a mean position accuracy of 7 cm, which was the best result compared to other tested methods. The authors of [58] proposed the use of the LSTM network for positioning objects with a fingerprinting method based on channel state information (CSI). CSI provides more information than the receiving signal strength indicator (RSSI) commonly used in Wi-Fi fingerprinting. The authors mapped high-dimensional amplitude and phase information to low-dimensional space using principal component analysis to reduce the input data size to the LSTM network, which predicts the position. The model the authors presented allows us to determine the object’s position with an average error of about 1 m (depending on, among others, the environment in which the research was carried out).

As presented, LSTM networks are widely used at different stages of object positioning. The research shows that they often give better results than other commonly used algorithms. No solution was found in which the LSTM network (which is commonly used for forecasting problems) was used for the position prediction of the object using displacement prediction.

The approach proposed in this article allows us to determine the moving object’s position with the use of an LSTM neural network based on data from UWB and IMU. There are the following contributions to this paper:

- The training neural network database was prepared, including movements in different directions and with different speeds and accelerations.
- The LSTM network architecture was introduced for object positioning based on UWB position (displacement) and IMU sensors.
- The prepared model was analyzed, and the accuracy of the position prediction was evaluated.
- The execution time of the prepared model was analyzed in the context of the displacement of the considered types of vehicles.

The paper is organized as follows. Section 2 describes the source of position errors and how much the displacement impacts it. Section 3 presents the system architecture and test scenarios prepared using the UWB simulator. Section 4 describes the structure of the LSTM network and pre-processing of the data. Section 5 describes the accuracy of the prepared model. Section 6 concludes the article.

## 2. Positioning of Moving Objects

Data pre-processing, correction, and filtration allow the removal of random system errors or measurement inaccuracies of the device or sensors used. The sensor measurements are affected by many factors, ranging from the manufacturing factors of the sensing device to the environmental factors present at the time of the measurement process (e.g., LOS and NLOS conditions).

Concentrating on the data from the UWB positioning system, the accuracy of the distances determined (collected in the first phase of the positioning) is affected by environmental conditions that can cause signal reflections or attenuation (e.g., a passing person or a passing vehicle between the UWB system nodes) or errors in distance measurement (e.g., rounding error which depends on the used microcontroller or clocks instability). The time of the whole positioning process should be known (starting from the ranging process, going through data acquisition, and ending with position calculation) to estimate the possible displacement of the moving object. It should be noted that in order to determine the position of the object on the plane (2D—two-dimensional), it is necessary to obtain at least three distances from the tag (a mobile UWB node) to the chosen reference points (stationary UWB nodes). However, processing the acquired distances is relatively small in relation to the time needed for data acquisition from the UWB system.

**Figure 1:**
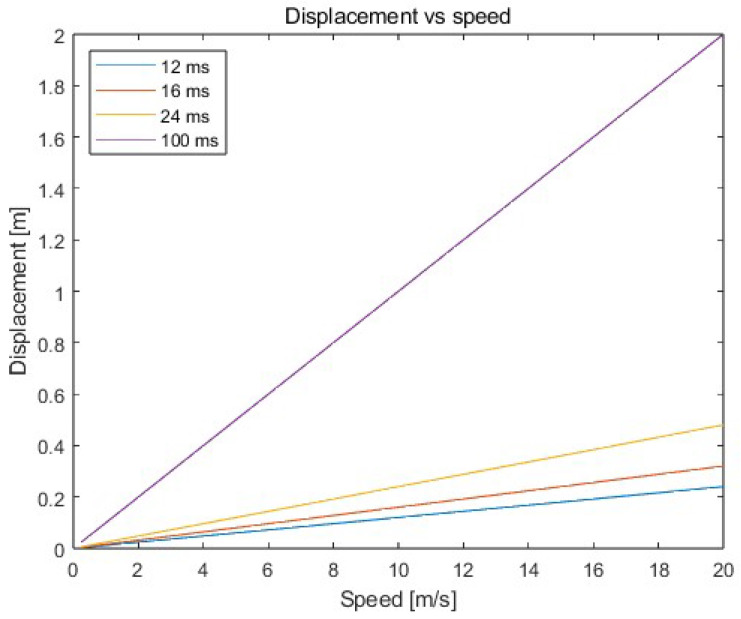
Displacement of the object depending on object speed and data acquisition time.

So, the most significant impact on the accuracy of the determined positions of the moving object is the time delay resulting from the ranging process between the tag placed on the moving object and the reference points. As the speed of the object increases and the acquisition time increases, the object’s displacement also increases—see Figure 1, according to Formula (1):

(1) $$d=v\cdot t_{p}$$

where d—displacement of the moving object; v—speed of the moving object; $t_{p}$—time needed to collect the required distances to determine the object’s position.

Therefore, the distances obtained from the system refer to positions from the past. On the market, various ready-to-use solutions for positioning objects using UWB technology can be found, which are characterized by different positioning frequency (which often depends on the size of the system—number of nodes) and accuracy. However, the accuracy of positioning systems often does not take the aspect of object movement into account (when the objects are constantly in motion—do not stop during the positioning process), and the accuracy of the system provided by the manufacturer applies, in fact, to stationary measurements.

An acquisition time of 100 ms results in a displacement of 1.4 m for a car moving at 14 m/s, see Figure 1. In such a situation, regardless of the accuracy of the UWB positioning system or the applied filtration on data, the moving object can lead to a dangerous situation (collisions with other traffic participants or elements of infrastructure). Therefore, it is necessary to predict the position of the object so that it is possible to detect dangerous situations. Additionally, predicting the position allows for determining the object’s position as accurately as possible (the calculated prediction of position corresponds to the current real position due to the fact that the object is in motion—the distances received are used to calculate the location at a given moment refer to past positions of the object).

## 3. System Description

Positioning of objects plays a vital role on a global scale (in the global frame of reference—the globe) and locally (in the local frame of reference covering a limited area, e.g., manoeuvering yards, intersections, warehouse halls, production halls). Information about the position in the local reference frame can be converted and used, for example, in a building diagram or a map of a town, which allows the location of objects in a broader sense. Systems and technologies that determine the object’s position differ from each other. They have their advantages and disadvantages, where the disadvantages of one technology should be outbalanced by the advantages of another or by appropriate data analysis.

**Figure 2:**
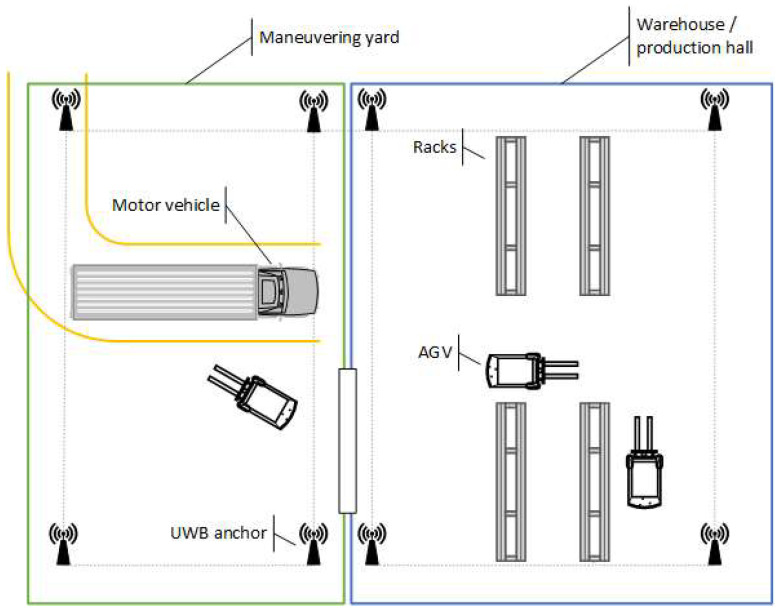
An example system scenario.

In this article, a positioning system consisting of a UWB and attitude and heading reference system (AHRS) that allows for positioning moving objects in a limited area is considered—an example system scenario is presented in Figure 2. The movement of AGVs (e.g., forklifts), which usually move at a speed of up to 2 m/s and acceleration of up to 2 m/s^2^, and motor vehicles (e.g., cars, motorbikes) moving in urban traffic at a speed of up to 14 m/s and acceleration of up to 10 m/s^2^ have been studied.

**Figure 3:**
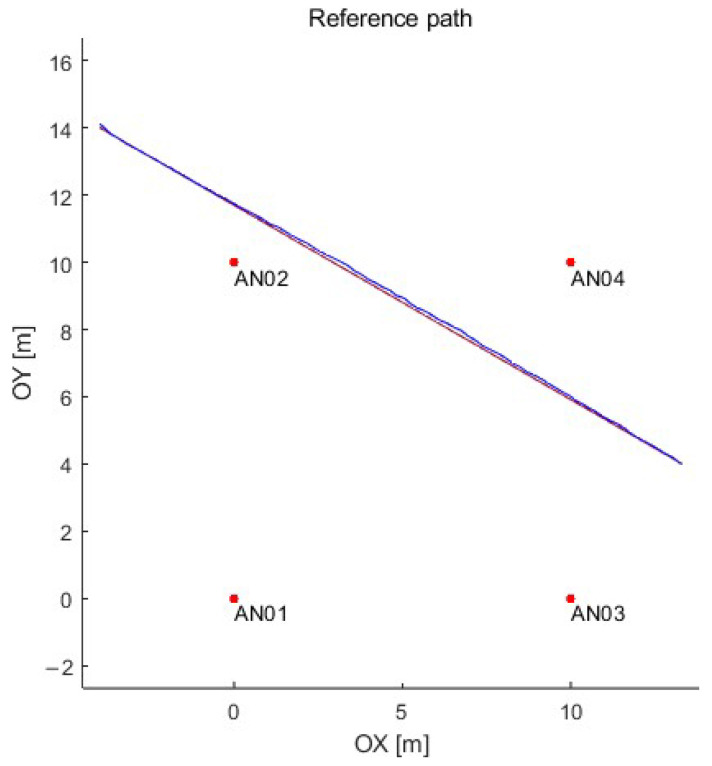
The simulation environment with sample path: anchors—red dots; reference path—red line; and path determined using the simulated UWB system—blue line.

This article proposes using the long short-term memory (LSTM) neural network to predict the object’s position based on data from the UWB (position in a 2D plane) and AHRS (acceleration, angular speed, and direction). Obtaining the ground truth (the reference position) for the UWB positioning system is complicated and often challenging if the movement of motor vehicles is considered. Therefore, the data set for artificial neural network training and testing was prepared in a simulation in the MATLAB computing platform. Data from the UWB system were generated using the authors’ simulator that reflects the operation of a real UWB system based on DW1000 (manufactured by Decawave—now part of Qorvo) modules (with a positioning time of 12 ms), which provide ground truth for further analysis [59]. On the other hand, the inertial unit was simulated using the simulator, which is built into the MATLAB computing platform, using the parameters of the Xsense MTI-670-DK inertial unit. The simulation environment with a sample path is presented in Figure 3. Four reference UWB nodes were arranged on a square plan of 10 m (no obstacles in the test stand) with the following coordinates (x [m], y [m]): anchor 1 (0, 0), anchor 2 (0, 10), anchor 3 (10, 0), anchor 4 (10, 10).

Simulation allows the preparation of a data set that is large enough to train the artificial neural network correctly and provide the reference positions. Moreover, the simulation allows for the preparation of test scenarios that are difficult to obtain in real conditions (e.g., enormous acceleration or speed) or the execution cost is very high.

Data were prepared for two variants of the object’s motion in a straight line:

- Variant A—uniform motion with speeds in the range of 0.25 m/s to 20 m/s, and
- Variant B—uniformly accelerated motion with an initial speed of 0 m/s and an acceleration in the range of 0.1 m/s^2^ to 10 m/s^2^.

The movement paths were carried out in such a way that they could reflect changes not only in the displacement value but also in terms of the direction of the object’s movement in the local frame of reference. The total number of test scenarios is 1716. The test parameters scenarios (variants A and B) are summarized in Table 1.

## 4. LSTM Network

**Figure 4:**
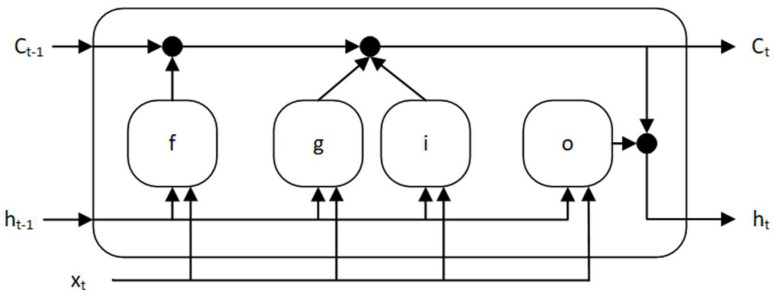
The memory cell structure of the LSTM neural network.

The LSTM network is a type of recurrent neural network that uses whole data sequences. A cell state contains information learned in the previous and current time steps. This type of network cell comprises three gates: input gate, output gate, and forget gate, which at every discrete time step add information to the memory cell or remove information from it [60,61,62,63]. The structure of the single memory cell is presented in Figure 4.

The $h_{t}$ vector is a hidden state of the cell (also called output) which corresponds to the short-term state (2), while the $c_{t}$ vector is a cell state corresponding to the long-term state (3). States of the cell are controlled using gates: the forget gate $f_{t}$, as the first, decides which long-term information will continue to pass the network—it decides which information stays and which gets deleted (4). Candidates to be added to the long-term state are selected using the cell candidate $g_{t}$ (5). New information is added by the input gate $i_{t}$ to the long-term state (6). In the end, a short-term state is created using the output gate $o_{t}$ (7):

(2) $$h_{t}=o_{t}⊙\sigma_{c}\left(c_{t}\right)$$

(3) $$c_{t}=f_{t}⊙c_{t-1}+i_{t}⊙g_{t}$$

(4) $$f_{t}=\sigma_{g}\left(W_{f}V_{t}+R_{f}h_{t-1}+b_{f}\right)$$

(5) $$g_{t}=\sigma_{c}\left(W_{g}V_{t}+R_{g}h_{t-1}+b_{g}\right)$$

(6) $$i_{t}=\sigma_{g}\left(W_{i}V_{t}+R_{i}h_{t-1}+b_{i}\right)$$

(7) $$o_{t}=\sigma_{g}\left(W_{o}V_{t}+R_{o}h_{t-1}+b_{o}\right)$$

where $W_{f},\;W_{g},\;W_{i},\;W_{o}$—input weights matrices of, respectively, forget gate, cell candidate, input gate, and output gate; $R_{f},\;R_{g},\;R_{i},\;R_{o}$—recurrent weights matrices of, respectively, forget gate, cell candidate, input gate, and output gate; $b_{f},\;\;b_{g},\;b_{i},\;b_{o}$—bias vector of, respectively forget gate, cell candidate, input gate, and output gate; $V_{t}$—input vector of the LSTM unit; $\sigma_{g}$—the gate activation function; $\sigma_{c}$—the state activation function; ⊙—denotes Hadamard product.

Input weights—$W_{f},\;W_{g},\;W_{i},\;W_{o}$, recurrent weights—$R_{f},\;R_{g},\;\;R_{i},\;R_{o}$, and biases—$b_{f},\;b_{g},\;b_{i},\;b_{o}$, are learned (tuned) during the training process of the network.

The activation function used to update the cell state and output (hidden state) is a hyperbolic tangent function (8), but the activation function applied to the gates is a sigmoid function (9) given as:

(8) $$\sigma_{c}=\frac{e^{2z}-1}{e^{2z}+1}$$

(9) $$\sigma_{g}=\frac{1}{1+e^{-z}}$$

where z—the summed weighted input (of the cell, gate).

The input data to the LSTM network—discussed in this article, are the displacements of the object determined using historical data from the UWB system—where the displacement of the object to the current position from the previous position is defined as displacement on the plane (*X* and *Y* axis) with the appropriate sign (10); and data obtained from the AHRS: accelerometer, gyroscope and magnetometer in *X*, *Y* and *Z* axes, at constant intervals. The data from the systems listed above form a vector of parameters—V (11), which is used in the neural network training process and then in the prediction of the position:

(10) $$\Delta P=\left\{\Delta x,\Delta y\right\}=\left\{x_{i}-x_{i-1},y_{i}-y_{i-1}\right\}$$

(11) $$V=\left\{v_{1},v_{2},\ldots ,v_{11}\right\}=\left\{\Delta x,\Delta y,a_{x},a_{y},a_{z},g_{x},g_{y},g_{z},m_{x},m_{y},m_{z}\right\}$$

where $v_{j}$—element of the neural network input, j∈1,2,…,11; Δx—displacement in the *X*-axis [m]; Δy—displacement in the *Y*-axis [m]; $a_{x},\;a_{y},\;a_{z}$—acceleration from the accelerometer, respectively, in the *X*, *Y*, and *Z*-axes [m/s^2^]; $g_{x},\;g_{y},\;g_{z}$—angular velocity from the gyroscope, respectively, in the *X*, *Y*, and *Z*-axes [rad/s]; and $m_{x},\;m_{y},\;m_{z}$—magnetic field from the magnetometer, respectively in the *X*, *Y*, and *Z*-axes [µT].

### 4.1. Pre-Processing of Data

Machine learning algorithms (especially those that use gradient descent methods for optimization) are, in most cases, sensitive to data (features) of different orders and ranges of values or units (12). Therefore, the data should be scaled before the training stage—in the pre-processing phase, and an appropriate scaling technique should be used, e.g., data normalization or standardization.

(12) $$\Theta_{l+1}=\Theta_{l}-\alpha \nabla E\left(\Theta_{l}\right)$$

where Θ—network parameters vector (weights and biases); α—learning rate; $E\left(\Theta \right)$—loss function, which is used to evaluate a machine learning algorithm.

The article used min-max normalization to scale the displacement (data obtained using UWB technology) and the data from inertial sensors (13). This method linearly converts the data to the desired interval $min_{x}^{\prime },max_{x}^{\prime }$. If the range from 0 to 1 is chosen, Equation (13) simplifies to (14):

(13) $$v_{j}^{\prime }=\frac{v_{j}-min_{v_{j}}}{max_{v_{j}}-min_{v_{j}}}\cdot \left(max_{v_{j}}^{\prime }-min_{v_{j}}^{\prime }\right)+min_{v_{j}}^{\prime }$$

(14) $$v_{j}{}^{\prime }=\frac{v_{j}-min_{v_{j}}}{max_{v_{j}}-min_{v_{j}}}$$

where $min_{v_{j}}$—the minimum value of the feature $v_{j}$; $max_{v_{j}}$—the maximum value of the feature $v_{j}$; $min_{v_{j}}^{\prime }$—the desired minimum value of the feature $v_{j}$; $max_{v_{j}}^{\prime }$—the desired maximum value of the feature $v_{j}$.

When the data used in the training of the artificial neural network are normalized, the values of the object displacement prediction (output of the network) are also normalized. Therefore, the output data from the LSTM network should be denormalized before further analysis using the previously adopted extreme values (15):

(15) $$v_{j}=v_{j}{}^{\prime }\cdot \left(max_{v_{j}}-min_{v_{j}}\right)+min_{v_{j}}$$

where $min_{v_{j}}$—the minimum value of the feature $v_{j}$; $max_{v_{j}}$—the maximum value of the feature $v_{j}$.

An overestimation or underestimation of the distances very often characterizes the UWB system. There are many reasons for this phenomenon, including the desynchronization of clocks between nodes, different accuracy of the clocks used, or the system configuration (delays). The correction mechanism is used in the pre-processing phase of data analysis to minimize the impact of the ranging error. The correction mechanism is based on mean bias error, expressed as a distance function using a second-degree polynomial. After receiving distances from the UWB system, the correction values are determined by which the distances obtained from the system should be reduced. Then, the corrected distances are used in the trilateration process (the trilateration method based on the simplex method is used in this research). The correction mechanism is described in detail in [64].

The estimation of the next position involves the use of data that could not describe similar cases but a whole set of different paths that should cover, to some extent, the possible situations (paths of movement) that may occur in the local frame of reference. It should be noted that in the proposed approach, successive displacements ΔP in the local coordinate system are used as the network’s input so that the network learns based on the displacement and not on absolute positions in the local frame of reference. So, the sequences (paths) are somewhat similar to each other (for a given speed and acceleration). This approach makes the system more universal and independent of the target coordinate system, which makes prediction possible on any section of the path along which the object is moving, regardless of its absolute position. It should also be mentioned that a finite number of straight lines can approximate an arc (bend).

### 4.2. Truncate Sequences

**Figure 5:**
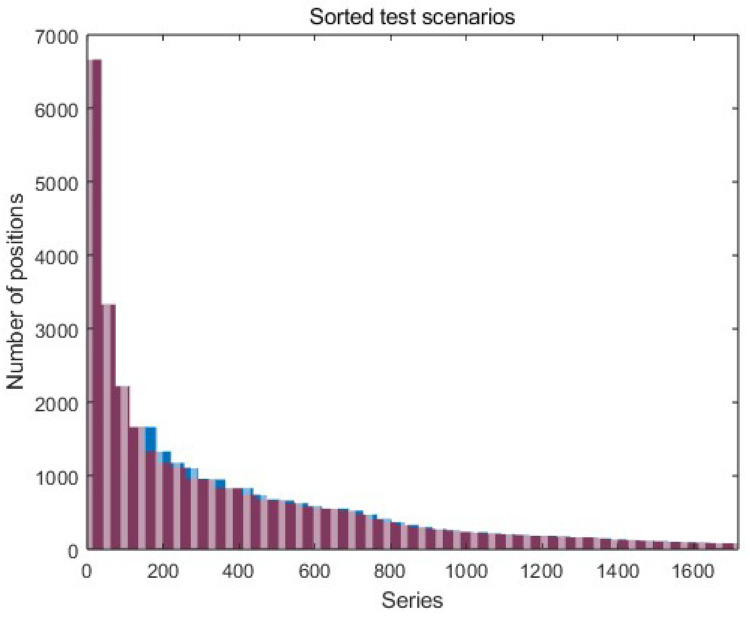
Sorted and trimmed data series (test scenarios)—the red indicates the series’ length, and the blue indicates removed (truncated) data from the series in the batch.

Due to a large number of measurement series (the average length of the series is 707 positions on the path) and the limited amount of computer memory, the data has to be divided into batches (a limited set of measurement series of the same length), which are sequentially put to the LSTM network. This solution updates the network parameters after each batch, and the data length (length of the path) between successive batches may vary. The data series in a single batch must be the same length as they all pass the network. For this purpose, after sorting the test scenarios and analyzing the length of all measurement series, it was decided that the best batch size is 37 series. Such a size of the batches does not require much interference in the series length (about 4% of all positions on the paths). It also allows for learning with the available operational memory. Batches consisting of 37 test scenarios are truncated to the shortest series in a given batch (red part of the graph in Figure 5). Another way is to extend the shorter series to the longest one. In this solution, data from the series are not lost; however, this procedure may introduce noise into the network, negatively affecting the predictive properties of the network.

### 4.3. Network Structure

**Figure 6:**
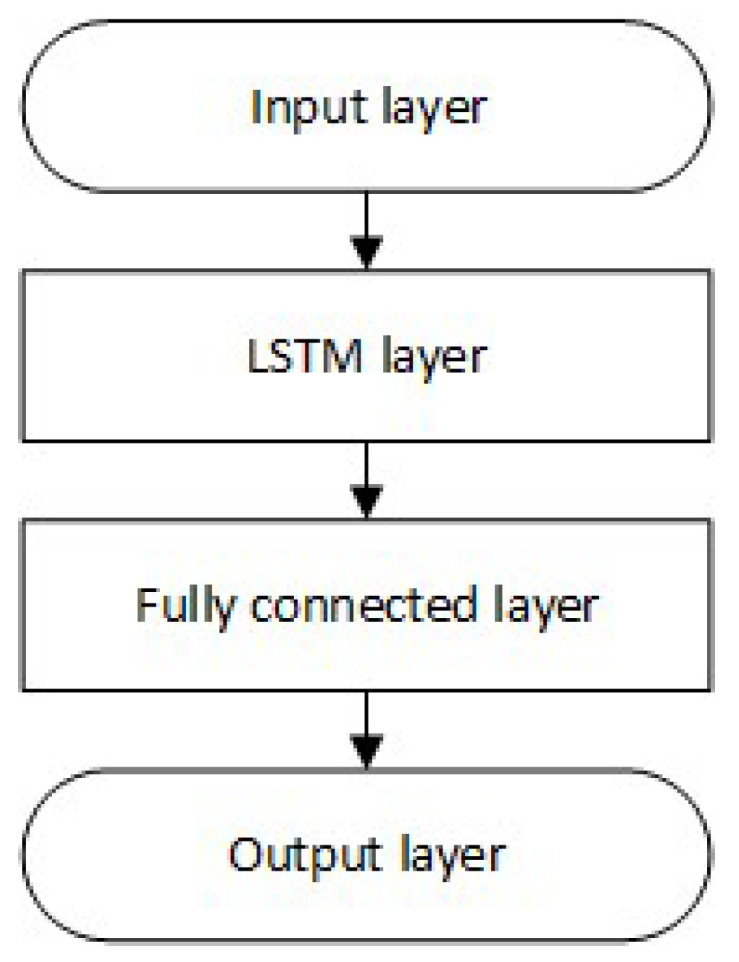
Structure of the LSTM neural network.

The research was carried out using the LSTM neural network with a different number of memory cells (the general structure of the network is shown in Figure 6). A network with one hidden layer was chosen because, in the problem of position prediction, an increase in error was noticed with the number of hidden layers while significantly extending the network learning time [57].

The input layer consists of 11 features (11), which are normalized using min-max normalization (see Section 4.1)—vector $V^{\prime }$ is obtained (16):

(16) $$V^{\prime }=\left\{\Delta x^{\prime },\;\Delta y^{\prime },a_{x}^{\prime },a_{y}^{\prime },a_{z}^{\prime },g_{x}^{\prime },g_{y}^{\prime },g_{z}^{\prime },m_{x}^{\prime },m_{y}^{\prime },m_{z}^{\prime }\right\}$$

where $\Delta x^{\prime }$—normalized displacement in the *X*-axis; $\Delta y^{\prime }$—normalized displacement in the *Y*-axis; $a_{x}^{\prime },a_{y}^{\prime },a_{z}^{\prime }$—normalized acceleration from the accelerometer, respectively, in the *X*, *Y*, and *Z*-axes; $g_{x}^{\prime },g_{y}^{\prime },g_{z}^{\prime }$—normalized angular velocity from the gyroscope, respectively, in the *X*, *Y*, and *Z*-axes; and $m_{x}^{\prime },m_{y}^{\prime },m_{z}^{\prime }$—normalized magnetic field from the magnetometer, respectively, in the *X*, *Y*, and *Z*-axes.

A hidden layer consisting of LSTM cells (networks with 20, 70, 100, and 150 memory elements in the hidden layer are analyzed), a fully connected layer, and an output layer fully connected with the last hidden layer (regression layer on which predicted displacement—output of the network, are obtained (17)).

(17) $$\Delta P^{\prime }=\left\{\Delta x_{p}^{\prime },\;\Delta y_{p}^{\prime }\right\}$$

where $\Delta x_{p}^{\prime }$—normalized predicted displacement in the *X*-axis; $\Delta y_{p}^{\prime }$—normalized predicted displacement in the *Y*-axis.

The learning process is based on the ADAM (adaptive moment estimation) optimizer.

## 5. Results

Prediction of the first position is performed on data known from the UWB system and inertial sensors (accelerometer, gyroscope, and magnetometer). However, each subsequent prediction is based on data from inertial sensors (available data from the system) and previous predictions (no data from the UWB system). Data associated with 20 previous positions were used to initialize the state of the trained network.

The performances of the trained neural networks were evaluated using the root mean square error (RMSE)—test set of data is a re-simulated data with the parameters described in Table 1. The RMSE of the prediction of ten positions depending on the number of memory cells broken down into the motion variant (A and B) is presented in Table 2. The starting position of the prediction is placed in 30%, 50%, and 80% of the movement of the twenty-meter path, which allows for the analysis of position accuracy at different stages of the object’s movement, covering various ranges of distances between the UWB anchors and tag (which affects the distance correction mechanism and thus the trilateration result). The memory cells of 150 reduce the error by about 2% for an increase in the number of memory cells by 50 compared to a network of 100 memory cells. Using a network with more than 100 memory cells does not bring many benefits but only increases the time needed to train the network and increases memory usage. For this reason, further results presented in this article should use the LSTM network, whose hidden layer consists of 100 memory cells. Such a network requires 44,600 weights (input weights: 4 (gates) × 100 (memory cells) × 11 (input features); recurrent weights: 4 × 100 × 100; output weights 2 (output of the network—displacements) × 100) and 402 biases (in the LSTM layer: 4 (gates) × 100 (memory cells); and in the fully connected layer: 2 (displacements)) to be tuned.

The accuracy of the prediction depending on the number of predicted positions is presented in Table 3. For randomly selected test scenarios from variants A and B, the prediction of the 1, 5, 10, 20, 40, 60, 80 and 100 positions were made. With the increase in the number of predicted positions, the prediction error increases from 6.53 cm for the first prediction to 94.25 cm for the 100th prediction of the position.

As mentioned above, the time of data processing influences the object’s displacement from the position where the first frame was exchanged to the place where the new position is available. On a personal computer with a six-core processor with a 3.6 GHz clock speed, the neural network initialization time is 1.7 ms, and the time for a single prediction is 1.9 ms (averaged values of all predicted positions). Network initialization should be performed when data from the UWB system are acquired (ranging process). Therefore, this time does not affect the displacement of the object. However, the prediction time affects the object’s displacement, which for a motor vehicle (14 m/s) results in a displacement of 2.7 cm, and for an AGV (2 m/s), a displacement of 0.4 cm. The displacement resulting from the prediction using the LSTM neural network is over six times smaller than the displacement resulting from the time of data acquisition from the UWB system (12 ms).

When the position prediction is carried out, the lack of data from the UWB system is simulated. As the LSTM neural network output gives the object displacement from the previous position to the next position, the last known position from the UWB system from which the next position can be calculated is required to determine the predicted position. Thus, the accuracy of the prediction also depends on the accuracy of the last known position received from the UWB system, from which subsequent positions are determined using the predicted displacement. So, pre-processing and data correction (of the distances from the UWB system) described in Section 4.1 are necessary and unavoidable.

**Figure 7:**
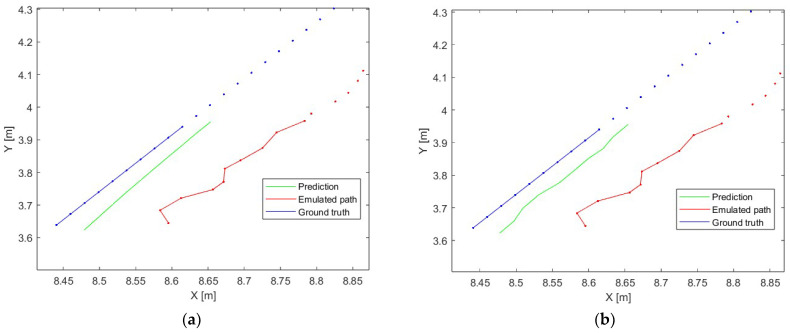
The example of the neural network prediction, (**a**)—no update from the UWB system over 10 positioning periods, (**b**)—update from the UWB system every positioning period.

An accurate comparison of the object position prediction error in variants A (divided into velocities) and B (divided into accelerations) was carried out for 10 position predictions (which corresponds to 120 ms when the acquisition time from the UWB system is 12 ms). The example of the prediction of 10 object positions is presented in Figure 7—the ground truth is the reference path of the movement (blue dots), the emulated path is the path determined using the UWB positioning system simulator (red dots), the prediction is the path obtained using presented data analysis and the prepared LSTM model (green line), the blue and red lines are the reference and simulated path, respectively, which correspond to the part of the movement which is predicted. Part (a) presents a case where there is no update from the UWB system over 10 positioning periods (no data from the UWB system over 120 ms), and part (b) presents a case where the network is updated every positioning period (data from the UWB system are available).

First, the accuracy of the UWB system for uniform motion is analyzed. The cumulative RMSE for ten object positions obtained from the UWB system in relation to the reference position is presented in Table 4. An increase in the average RMSE can be observed with each successive prediction, remaining below 5 cm until the seventh position prediction (which for a motor vehicle (14 m/s) results in a displacement of 1.18 m, and for an AGV (2 m/s) a displacement of 17 cm). It should be noted that for low speeds with a position acquisition frequency of 12 ms, the error is most affected by the inaccuracy of the UWB system, not the displacement of the object—for the speed of 0.5 m/s, the object will move only by 6 mm, where the accuracy of the presented system can be assumed at 10 cm.

Next, the accuracy of the UWB system for uniformly accelerated motion is analyzed. The cumulative RMSE for ten object positions obtained from the UWB system in relation to the reference position (obtained from the UWB system simulator) is presented in Table 5. Since the accelerated motion started from v_0_ = 0 [m/s], the displacements at the beginning of the movement between successive positions are small. The most significant impact on the RMSE is the inaccuracy resulting from the trilateration algorithm, not the object’s displacement. The system’s accuracy ranges from 4.5 cm to 5.3 cm. There is a visible trend towards increasing RMSE with increasing acceleration, which is associated with greater dynamics of changes in displacement. However, the average RMSE value is about 5 cm, which is comparable to the results obtained for uniform motion.

The obtained accuracy of the predicted positions for both the AGV and the motor vehicle is sufficient to allow uninterrupted positioning of the object during a short period (up to 120 ms) of signal loss from the UWB system and to predict the next position of the object. For AGVs, which usually move at a speed of up to 2 m/s and acceleration up to 2 m/s^2^, the RMSE value of the prediction of a path consisting of 10 positions is 4.9 cm for uniform motion, and 4.3 cm uniformly accelerated motion (according to extreme values). For motor vehicles moving in urban traffic at speeds up to 14 m/s and accelerations up to 10 m/s^2^, the RMSE value for the prediction of a path consisting of 10 positions is 7.3 cm for uniform motion and 6.8 cm for uniformly accelerated motion (according to extreme values).

**Figure 8:**
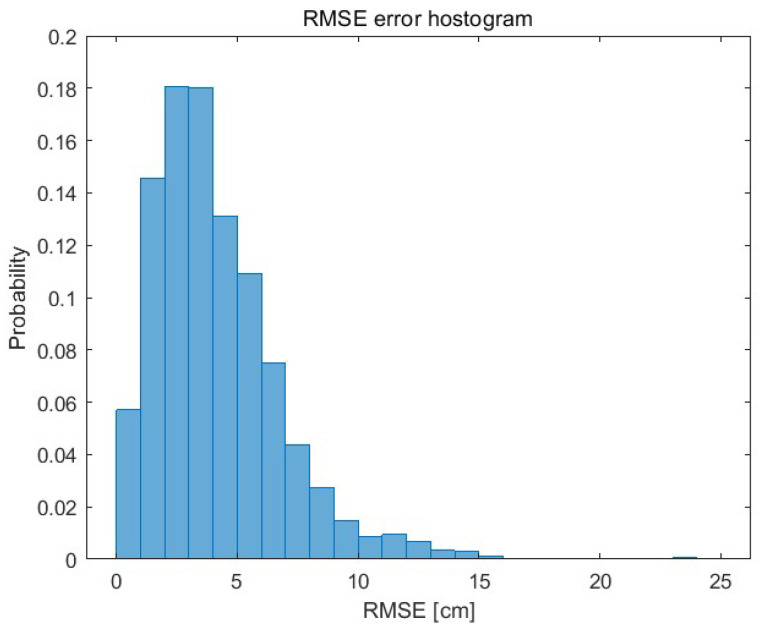
The RMSE histogram of the 10 position prediction.

**Figure 9:**
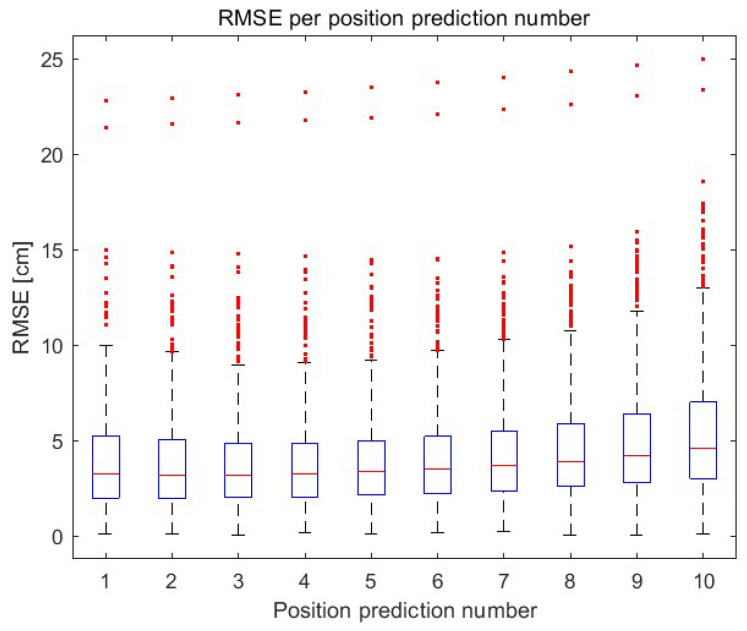
The RMSE up to the 10th prediction.

The histogram showing the RMSE of each (independently) prediction of 10 object positions is shown in Figure 8. The RMSE of each subsequent predicted object position (up to the 10th prediction) is presented using a boxplot in Figure 9. The 75th percentile is 7 cm (10th prediction of the position).

The prepared model was also tested in different test scenarios. As previously mentioned, four reference UWB nodes were arranged on a square plan of 10 m (no obstacles in the test stand). The movements of the object were simulated (using the same UWB simulator as in scenarios A and B) not only in straight lines but also in arcs in two variants:

- Variant C—uniform motion with speeds in the range of 0.25 m/s to 20 m/s, and
- Variant D—uniformly accelerated motion with initial speeds in the range of 0 m/s to 18 m/s and an acceleration in the range of 0.1 m/s^2^ to 10 m/s^2^.

The movement paths reflect the changes in the displacement value and the direction of the object’s movement for both lines and arcs (in two turning variants: clockwise and counterclockwise). The total number of test scenarios is 23,572.

The influence of a larger (30, 40, 50, 80) and smaller (10) number of V vectors used to initialize the LSTM network was checked (in the previous considerations, 20 vectors V were used). The RMSE of the prediction of ten positions depending on the number of previous positions used to initialize the state of the trained network for the motion variants C and D is presented in Table 6 and Table 7, respectively. The first row shows the RMSE of the raw data—the average RMSE of RAW data for variant C is 10.4 cm and 9.6 for variant D. For variants C and D, initialization with 10 previous positions gives a 6% greater error in relation to raw data; while in relation to initialization with 20 previous positions, the error is greater by 70% for variant C and 31% for variant D. For variant C, 20 previous positions allow to achieve error less than 10 cm up to 10 position predictions. However, for variant D to achieve an error of less than 10 cm up to 10 position predictions, the number of previous positions has to be doubled (up to 40).

As can be seen, the increase in the number of positions used for network initialization positively affects prediction accuracy. However, the greatest increase in accuracy is visible for 40 initialization vectors. The prepared object position prediction system allows us to effectively predict 10 object positions in scenarios that did not occur in the training set. In addition, a position prediction error is often lower (starting from 20 initialization vectors—starting from the third row in Table 6 and Table 7) than a position calculated directly from UWB data (the first row in Table 6 and Table 7—RAW)—error less than 10 cm up to 10 position predictions.

**Figure 10:**
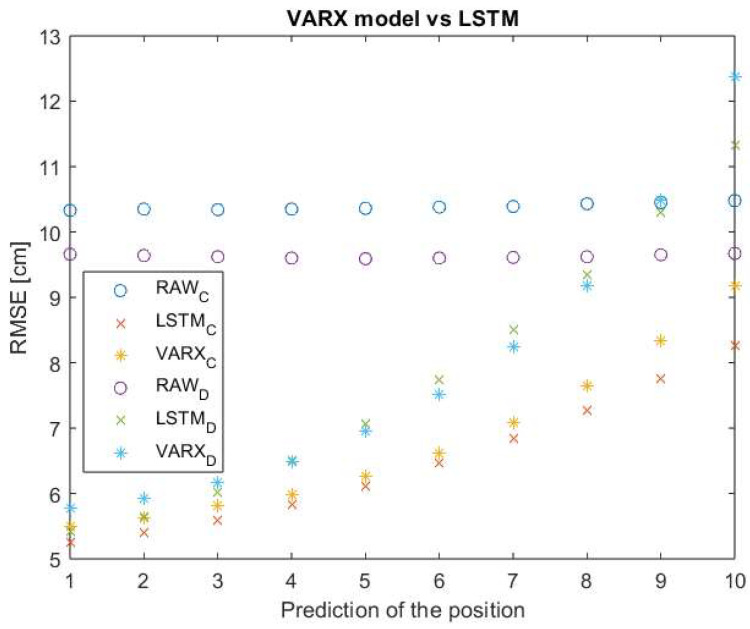
The RMSE up to the 10th prediction—VARX and LSTM.

For randomly selected test scenarios from variants C and D, 10 positions were predicted using the VARX model with a lag of 3 and the presented LSTM network—initialization with 20 previous positions. Figure 10 shows the RMSE for simulated data (circles), position predictions using LSTM (crosses), and the VARX model (asterisks).

On average, the RMSE of the LSTM network is 3% lower than for the VARX model. It should be noted that the time needed to determine a single prediction in the VARX model is 8.7 ms, 4.5 times greater than in the case of the LSTM network (which will result in 4.5 times greater displacement of the object during the prediction).

## 6. Conclusions

The accuracy of the position determination depends on the accuracy of the determined distances between the nodes in the network and the appropriate pre-processing. The computing units used in the vehicles (e.g., cars, AGVs) are computationally efficient, so the accuracy of the determined position is mainly affected by the delay connected with the time needed to measure the distance between the UWB nodes in the network (ranging process) and to make the position available for other purposes. Reduction in data processing and acquisition time is not possible, but it is possible to determine the position prediction based on historical data using the LSTM neural network. Using filters with a moving window for real-time systems is not recommended, considering the continuous movement of the objects.

The conducted experiments and comparisons prove that the prediction of the object’s position is possible using the prepared LSTM neural network with only one hidden layer with high accuracy—it enables the determination of the object’s position with an accuracy of less than 10 cm up to 10 positions (for both dataset of test scenarios) which correspond to 120 ms of continuous positioning of a moving object (which gives 24 cm of the driven distance for AGVs and 1,68 m for motor vehicles). The number of predicted positions can be increased, considering the simultaneous increase in the prediction error. It should be borne in mind that the accuracy of a larger number of predictions can be affected by a rapid change in the object’s motion parameters (direction, acceleration). Therefore, to maintain the system’s high accuracy, the network requires periodic initialization (e.g., with each subsequent data packet from the UWB system).

## Figures and Tables

**Table 1:** Test scenarios summary.

Test Parameter	Value
Acceleration [m/s^2^]	0; 0.1; 0.2; 0.3; 0.4; 0.5; 0.6; 0.7; 0.8; 0.9; 1; 2; 3; 4; 5; 6; 7; 8; 9; 10
Initial speed [m/s]	0; 0.25; 0.5; 0.75; 1; 1.25; 1.5; 1.75; 2; 2.5; 3; 3.5; 4; 5; 6; 7; 8; 9; 10; 11; 12; 13; 14; 15; 16; 17; 18; 19; 20
Direction of movement [rad]	$\left\{0,\;\pi ,2\pi \right\}\pm \left\{\frac{\pi }{10},\frac{\pi }{9},\frac{\pi }{8},\frac{\pi }{7},\frac{\pi }{6},\frac{\pi }{5},\frac{\pi }{4},\frac{\pi }{3}\right\}$
Path length [m]	20

**Table 2:** RMSE of the 10 prediction positions of the moving object at different stages of the path.

Number of Memory Cells	Variant of Movement	RMSE [cm]			
		80%	50%	30%	Avg
20	A	6.23	6.40	9.46	7.36
	B	5.76	6.49	9.81	7.36
70	A	5.76	5.22	8.36	6.45
	B	5.13	6.21	9.32	6.89
100	A	5.68	4.57	7.77	6.01
	B	4.49	5.75	9.29	6.51
150	A	5.78	4.37	7.31	5.82
	B	4.14	5.10	9.45	6.23

**Table 3:** Cumulative RMSE of the object position predictions.

	Number of Predictions							
	1	5	10	20	40	60	80	100
RMSE [cm]	6.53	6.82	7.54	10.44	21.49	39.12	63.31	94.25

**Table 4:** The RMSE of the position prediction for uniform motion.

v [m/s]	RMSE [cm]									
	1	2	3	4	5	6	7	8	9	10
0.25	4.90	4.98	5.06	5.14	5.24	5.35	5.47	5.60	5.74	5.89
0.5	3.71	3.76	3.81	3.87	3.93	4.01	4.10	4.20	4.32	4.44
0.75	3.40	3.45	3.48	3.54	3.60	3.69	3.79	3.90	4.02	4.17
1	2.63	2.69	2.74	2.79	2.87	2.95	3.05	3.16	3.29	3.42
1.25	2.96	3.00	3.04	3.08	3.13	3.20	3.27	3.35	3.44	3.54
1.5	3.20	3.21	3.24	3.28	3.33	3.39	3.46	3.54	3.63	3.74
1.75	3.33	3.38	3.46	3.57	3.72	3.89	4.09	4.32	4.57	4.85
2	3.22	3.30	3.42	3.57	3.72	3.90	4.11	4.34	4.58	4.86
2.5	2.59	2.67	2.74	2.84	2.96	3.12	3.30	3.51	3.74	3.98
3	2.56	2.60	2.67	2.75	2.86	3.00	3.17	3.39	3.63	3.89
3.5	2.99	2.97	2.97	3.02	3.11	3.23	3.38	3.56	3.76	3.99
4	3.13	3.13	3.18	3.28	3.43	3.64	3.88	4.16	4.47	4.82
5	3.06	2.98	2.95	2.99	3.11	3.29	3.53	3.83	4.16	4.54
6	3.19	3.22	3.31	3.49	3.74	4.06	4.42	4.83	5.26	5.73
7	3.33	3.34	3.42	3.62	3.91	4.29	4.71	5.19	5.70	6.25
8	3.30	3.19	3.23	3.46	3.77	4.20	4.68	5.23	5.81	6.45
9	3.99	3.77	3.70	3.77	3.94	4.22	4.58	5.00	5.47	6.01
10	4.36	4.09	3.96	3.98	4.15	4.44	4.81	5.28	5.80	6.38
11	4.67	4.31	4.12	4.11	4.26	4.56	4.96	5.43	6.00	6.62
12	5.90	5.40	4.99	4.73	4.63	4.71	4.93	5.29	5.74	6.27
13	6.29	5.89	5.55	5.38	5.35	5.46	5.68	6.01	6.40	6.87
14	6.38	5.80	5.37	5.14	5.11	5.27	5.61	6.06	6.63	7.29
15	6.79	6.20	5.75	5.47	5.41	5.53	5.82	6.24	6.76	7.35
16	7.54	7.04	6.63	6.37	6.24	6.26	6.41	6.66	7.01	7.44
17	8.39	7.81	7.27	6.85	6.59	6.50	6.55	6.76	7.06	7.47
18	9.40	8.75	8.23	7.77	7.41	7.17	7.07	7.12	7.30	7.60
19	10.02	9.48	8.91	8.44	8.07	7.82	7.70	7.71	7.84	8.08
20	10.08	9.41	8.95	8.45	8.03	7.68	7.41	7.24	7.17	7.21
Avg	4.83	4.64	4.50	4.45	4.49	4.60	4.78	5.03	5.33	5.68

**Table 5:** The RMSE of the position prediction for uniformly accelerated motion.

a [m/s^2^]	RMSE [cm]									
	1	2	3	4	5	6	7	8	9	10
0.1	2.74	2.76	2.77	2.79	2.84	2.91	3.00	3.12	3.25	3.39
0.2	2.95	2.90	2.87	2.83	2.82	2.83	2.87	2.92	2.99	3.08
0.3	2.86	2.86	2.92	3.02	3.16	3.33	3.54	3.75	4.01	4.27
0.4	3.93	3.92	3.92	3.91	3.91	3.91	3.93	3.96	4.00	4.08
0.5	3.20	3.12	3.04	2.97	2.92	2.90	2.89	2.93	2.99	3.08
0.6	3.67	3.57	3.49	3.44	3.40	3.37	3.36	3.38	3.42	3.49
0.7	3.62	3.49	3.39	3.29	3.20	3.12	3.06	3.03	3.01	3.01
0.8	3.74	3.70	3.63	3.56	3.51	3.49	3.48	3.51	3.55	3.62
0.9	3.97	3.86	3.76	3.69	3.62	3.58	3.56	3.58	3.64	3.72
1	4.21	4.10	3.99	3.90	3.82	3.77	3.75	3.75	3.79	3.85
2	5.19	5.00	4.82	4.67	4.55	4.44	4.37	4.31	4.29	4.30
3	5.79	5.60	5.40	5.22	5.05	4.91	4.81	4.74	4.72	4.75
4	7.06	6.95	6.85	6.72	6.60	6.48	6.36	6.25	6.16	6.08
5	6.44	6.21	5.94	5.69	5.43	5.21	5.02	4.88	4.78	4.76
6	7.23	6.93	6.63	6.33	6.09	5.85	5.67	5.54	5.46	5.44
7	7.61	7.27	6.94	6.62	6.28	5.97	5.70	5.47	5.31	5.22
8	8.33	8.07	7.74	7.43	7.14	6.84	6.59	6.38	6.24	6.16
9	8.76	8.49	8.16	7.83	7.48	7.15	6.85	6.58	6.38	6.24
10	9.49	9.21	8.88	8.58	8.25	7.92	7.61	7.31	7.05	6.83
Avg	5.30	5.16	5.01	4.87	4.74	4.63	4.55	4.49	4.48	4.49

**Table 6:** The RMSE of the position prediction for variant C—lines and arcs.

Number of V	RMSE [cm]									
	1	2	3	4	5	6	7	8	9	10
RAW	10.33	10.35	10.34	10.35	10.36	10.38	10.39	10.43	10.45	10.48
10	7.08	7.80	8.64	9.50	10.40	11.33	12.28	13.27	14.28	15.32
20	5.26	5.40	5.59	5.83	6.12	6.46	6.84	7.27	7.75	8.27
30	4.99	5.07	5.20	5.39	5.64	5.95	6.32	6.75	7.24	7.80
40	4.88	4.85	4.86	4.92	5.03	5.20	5.42	5.70	6.03	6.42
50	4.88	4.83	4.80	4.81	4.88	4.99	5.16	5.39	5.66	5.99
80	4.40	4.38	4.39	4.44	4.53	4.66	4.84	5.06	5.32	5.63

**Table 7:** The RMSE of the position prediction for variant D—lines and arcs.

Number of V	RMSE [cm]									
	1	2	3	4	5	6	7	8	9	10
RAW	9.66	9.64	9.62	9.60	9.59	9.60	9.61	9.62	9.65	9.67
10	6.36	6.89	7.62	8.43	9.34	10.32	11.39	12.53	13.74	15.03
20	5.42	5.64	6.02	6.50	7.07	7.74	8.50	9.35	10.29	11.32
30	5.35	5.55	5.92	6.39	6.96	7.63	8.40	9.27	10.24	11.30
40	5.15	5.18	5.34	5.57	5.89	6.29	6.77	7.34	7.99	8.72
50	5.24	5.18	5.22	5.33	5.52	5.77	6.11	6.52	7.01	7.57
80	5.22	5.08	5.05	5.07	5.15	5.28	5.48	5.75	6.07	6.47

## Data Availability

The data presented in this study are available on request from the corresponding author.

## Abbreviations

d	Displacement of the moving object
v	Speed of the moving object
$t_{p}$	Time needed to collect the required distances to determine the object’s position
$h_{t}$	Hidden state of the cell
$c_{t}$	Long-term state
$f_{t}$	Forget gate
$g_{t}$	Cell candidate
$W_{f},W_{g},W_{i},W_{o}$	Input weights of, respectively, forget gate, cell candidate, input gate, and output gate
$R_{f},R_{g},R_{i},R_{o}$	Recurrent weights of, respectively, forget gate, cell candidate, input gate, and output gate
$b_{f},b_{g},b_{i},b_{o}$	Bias of, respectively, forget gate, cell candidate, input gate, and output gate
$V_{t}$	Input vector of the LSTM unit
$\sigma_{g}$	Gate activation function
$\sigma_{c}$	The state activation function
z	The summed weighted input of the cell
$v_{j}$	Element of the neural network input
Δx	Displacement in the *X*-axis
Δy	Displacement in the *Y*-axis
$a_{x},a_{y},a_{z}$	Acceleration from the accelerometer, respectively, in the *X*, *Y*, and *Z*-axes
$g_{x},g_{y},g_{z}$	Angular velocity from the gyroscope, respectively, in the *X*, *Y*, and *Z*-axes
$m_{x},m_{y},m_{z}$	Magnetic field from the magnetometer, respectively, in the *X*, *Y*, and *Z*-axes
Θ	Network parameters vector
α	Learning rate
$E\left(\Theta \right)$	Loss function
